# Thermal imaging and deep learning-based fit-checking for respiratory protection

**DOI:** 10.1038/s41598-024-52999-0

**Published:** 2024-10-17

**Authors:** Hyunjin Kim, Tong Min Kim, Sae Won Choi, Taehoon Ko

**Affiliations:** 1 Department of Medical Sciences, College of Medicine, The Catholic University of Korea, 222 Banpo-daero, Seocho-gu, Seoul, 06591 Republic of Korea; 2 Department of Medical Informatics, College of Medicine, The Catholic University of Korea, 222 Banpo-daero, Seocho-gu, Seoul, 06591 Republic of Korea; 3 CMC Institute for Basic Medical Science, The Catholic Medical Center of The Catholic University of Korea, 222 Banpo-daero, Seocho-gu, Seoul, 06591 Republic of Korea; 4 Department of Emergency Medicine, Veterans Health Service Medical Center, 53 Jinhwangdo-ro 61-gil, Gangdong-gu, Seoul, 05368 Republic of Korea

**Keywords:** Health care, Health occupations

## Abstract

This study develops an artificial intelligence model to quickly and easily determine correct mask-wearing in real time using thermal videos that ascertained temperature changes caused by air trapped inside the mask. Five types of masks approved by the Korean Ministry of Food and Drug Safety were worn in four different ways across 50 participants, generating 5000 videos. The results showed that 3DCNN outperformed ConvLSTM in both binary and multi-classification for mask wearing methods, with the highest AUROC of 0.986 for multi-classification. Each mask type scored AUROC values > 0.9, with KF-AD being the best classified. This improved use of thermal imaging and deep learning for mask fit-checking could be useful in high-risk environments. It can be applied to various mask types, which enables easy generalizability and advantages in public and occupational health and healthcare system.

## Introduction

Exposure to particulate matter can cause serious health risks^1,2^. Particulate matter, which constitutes an inhalable particle in the air, is classified based on size into PM10 and PM2.5 (particles with a diameter < 10 and < 2.5 μm, respectively)^3^. PM10 and PM2.5 can easily penetrate the lungs and bloodstream and thereby cause or exacerbate health problems, such as respiratory and cardiovascular diseases^3,4^. In 2013, the International Agency for Research on Cancer (IARC) designated particulate matter as a Group 1 carcinogen that was a substance harmful to the human body^5^. In particular, healthcare workers face a high risk of exposure to particulate matter due to their work environment. This exposure often occurs when using equipment like lasers and electrocautery, or during medical procedures such as tissue cutting and cauterization^6^. Moreover, particulate matter can be generated when many people from outside the hospital are sequestered within the hospital^7^. Besides healthcare workers in hospitals, individuals in specific occupational health environments, such as factories and mines, are more likely to experience occupational exposure to particulate matter.

Healthcare workers regularly come into contact with diseases and infections. Due to the nature of their work, healthcare workers are exposed to various viruses, and their risk of infection increases significantly during epidemics such as the severe acute respiratory syndrome (SARS)^8^. The recent coronavirus disease (COVID-19) pandemic caused infection and deaths of numerous healthcare workers, further emphasizing this risk^9^. As hospitals accommodate many patients and contain many sources of infection, it is important to implement stringent infection-control measures to protect the health and well-being of healthcare workers.

Masks are used as a major solution to these concerns. Masks easily protect the wearer and people around them from infection and block the entry of harmful substances, such as fine particles and dust in the air^10,11^. The Korean Ministry of Food and Drug Safety (MFDS) recommends the use of masks approved as quasi-drugs to protect the respiratory tract from pathogens and fine particles^12,13^. Thus, a mask is a non-pharmacological intervention that is used to prevent diseases without directly affecting the human body. The mask blocks fine particles and droplets and is reliable, as its safety in use has been confirmed^14^. The Ministry of Employment and Labor of Korea mandates the provision of masks at work sites with high fine-dust concentrations per the Occupational Safety and Health Act, where the use of Korean MFDS-certified quasi-drug masks is mandated^12^.

Wearing a mask properly is just as important as deciding to wear one^15^. The US Occupational Safety and Health Administration (OSHA) and the Korea Occupational Safety and Health Agency (KOSHA) mandate an annual respiratory protection fit test to ensure correct mask-wearing^16^. A fit test ensures that the mask fits effectively against the face. It consists of a Qualitative Fit Test (QLFT) and a Quantitative Fit Test (QNFT). The QLFT uses the five senses, including human smell and taste, to determine whether contaminants are penetrating the inside of the mask, which can be unreliable due to the tester's subjective opinion. QNFT is a method that measures the concentration or pressure of aerosols in the air to calculate the degree of leakage as a fit factor. This is done in conjunction with an exercise regime^16^. The fit test takes a total of 15 to 20 min per person. It requires various materials and equipment such as test agents (e.g., saccharin), test hoods, etc. Therefore, it is difficult to perform it quickly in real time. Also, it is hard to conduct many tests because the cost varies greatly depending on the number of tests^17,18^.

Accordingly, methods have been proposed to execute the fit test more efficiently. Nelson et al. attempted to reduce the test duration to 15 s instead of 1 min for the QLFT by using bitter (Bitrex™) aerosol^19^. Zhuang et al. tried to decrease the seven execution systems performed in the fit test to three^20^. Nevertheless, a study wherein the N95 fit test was conducted on 6287 people in 2021 showed that considerable time and human and material resources were consumed and thereby conferred limitations^21^.

Unlike conventional inspections, which are complicated by the diverse requisite materials and steps, thermal imaging cameras focus only on the relative temperature^22^. Therefore, studies on breast cancer and image diagnosis have confirmed the usefulness of thermal imaging in a healthcare environment by detecting and visualizing temperature differences based on infrared images^23,24^. Moreover, Harber et al., using infrared imaging, demonstrated the detection of air leakage from the mask. Expressing the leakage detected by infrared imaging as a numerical value through an expert revealed the possibility of supplementing the disadvantage wherein a mask that passed the QNFT, expressed numerically, could be inaccurate when worn^25^. Siah et al. suggested a fit-checking method using infrared images and deep learning technology for respiratory protective devices that focused on the temperature change due to air leakage at the edge of the mask in the captured image^8^. Therefore, it is necessary to photograph the face from various angles to check the edge of the mask, and this method is not easy to use it in real-time in a continuously dynamic environment.

The primary contribution of this study is the development of an innovative artificial intelligence model that can quickly and accurately determine if a mask is worn properly in real-time, using thermal imaging. This approach leverages the temperature changes caused by air trapped inside the mask. It offers an effective solution to ensuring proper mask usage, crucial for the safety of healthcare workers and others in high-risk environments. We hypothesized that correct mask-wearing reduces air leakage and increases the temperature inside the mask^26,27^, a theory we tested with five different mask types. This study evaluates multiple mask types, providing a comprehensive understanding of mask efficiency across various designs. Furthermore, our method focuses on relative temperature changes, making it adaptable to masks of different shapes and designs. This versatility allows our model to help in respiratory protection, which can contribute to occupational health and safety.

This paper is organized as ollows. The next section, "Materials and methods", describes the data collection and analysis processes in detail. "Results" summarizes the classification outcomes using deep learning, and "Discussion" covers their interpretation. Finally, "Conclusions" presents this research's expected impact and future possibilities.

## Materials and methods

### Participants

This study was conducted with 50 healthy male and female adult participants (age range 19–65 years). The study did not include individuals with respiratory diseases who found mask-wearing uncomfortable. All regulatory requirements (ethics approval) were obtained from the Institutional Review Board before the commencement of the study, and participants were recruited only after obtaining informed consent. All data were collected anonymously, and no personally identifiable information (e.g., name and contact information) was obtained. In addition, participants were informed that participation in the study was voluntary, and they were allowed to withdraw from the study at any time if necessary.

### Data collection

The thermal imaging camera used is the FLIR ONE Pro. It has a frame rate of 8.7 Hz, and a visual resolution of 1440 × 1080. It can be conveniently used by simply attaching it to a mobile phone—an iPhone Xs in this study. The recording was conducted in an environment with a stable room temperature of 20 °C. Throughout the entire recording space, including areas near the camera and the subject, we ensured the absence of any heat sources or objects that could disrupt accurate temperature measurements.

Five types of masks (surgical mask, KF-Anti-Droplet [KF-AD], KF80, KF94, and KF99) were used in this study. They are approved as quasi-drugs by the Korean MFDS. The Korean Filter (KF) is a standard that has been newly established by the Korean MFDS based on the existing European mask standards^28^. KF80 can filter out more than 80% of fine particles with an average size of 0.6 μm, and KF94 and KF99 can filter out more than 94% and 99% of fine particles with an average size of 0.4 μm, respectively. KF-AD is a mask used to prevent droplet (saliva) infection, and a surgical mask is a mask that is worn during medical examination, treatment, or surgery^13,14,29^. KF94 is considered of a similar grade as the US NIOSH N95 and European FFP2 masks^30^, and KF80 is considered to be of a similar grade as FFP1^31^.

**Figure 1:**
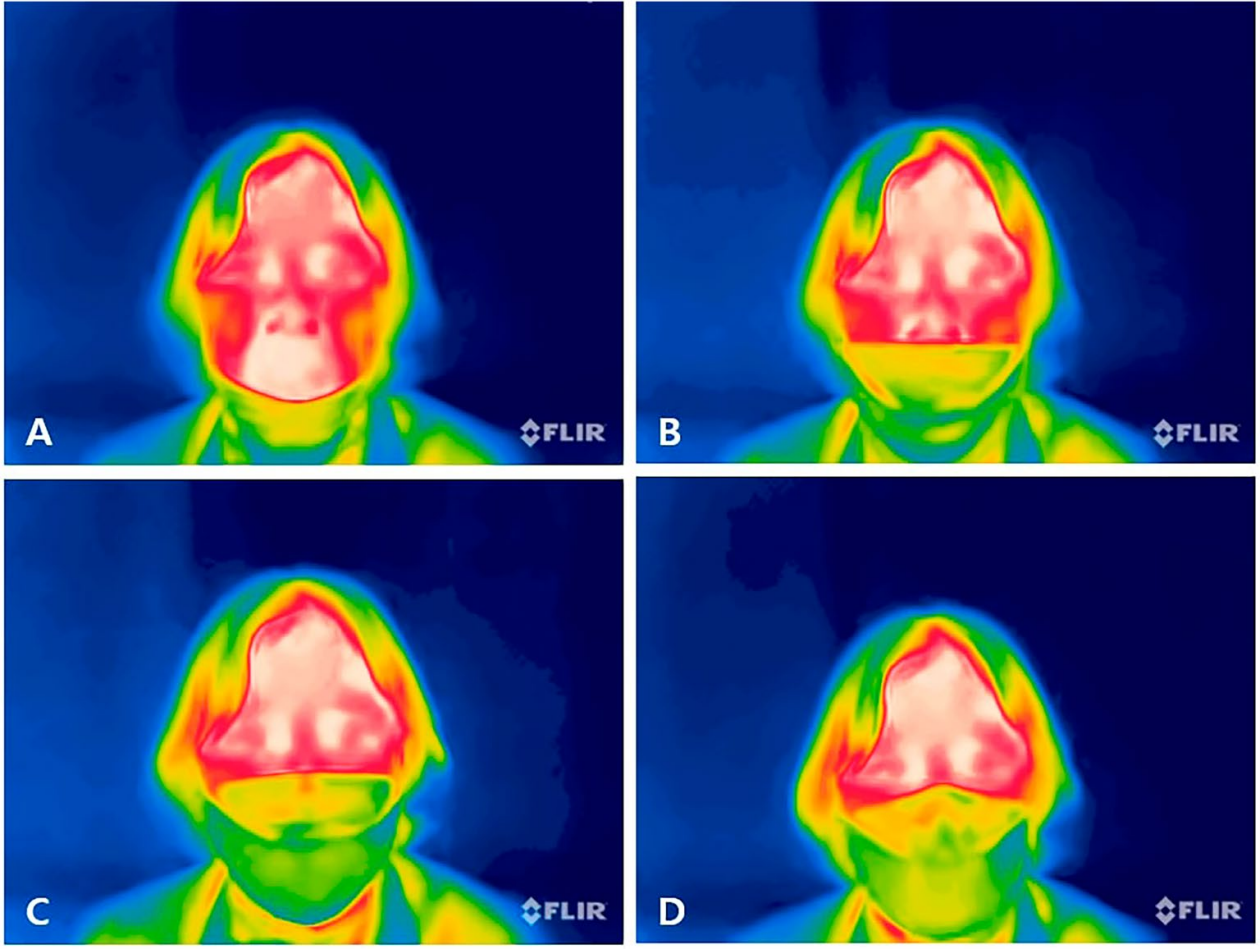
Four methods of mask-wearing: (**A**) wearing a mask on the chin to expose the nose and mouth; (**B**) lowering the mask under the nose and covering only the mouth; (**C**) covering the nose without the nose wire being tightly attached, thereby letting air leak from the side of the mask; (**D**) wearing the mask properly and securely.

Each participant sat in a chair facing the camera, with the distance between the camera and their face fixed at 35 cm. This setup aimed to include only the face in the video as much as possible. Based on the normal adult respiratory rate of 12–20 breaths per minute^32^, a video of a person taking one breath for 4 s while wearing each type of mask was recorded. Breathing was performed alternately between inhalation and exhalation at 2-s intervals using a metronome. There were four methods to wear a mask, according to the area covered by the mask (Fig. 1A–D): wearing a mask on the chin to expose the nose and mouth (Fig. 1A); lowering the mask under the nose and covering only the mouth (Fig. 1B); covering the nose without the nose wire being tightly attached and letting air leak from the side of the mask (Fig. 1C); wearing the mask properly and tightly (Fig. 1D). Figure 1A–C show improper methods of mask-wearing, and only Fig. 1D shows the proper method of wearing a mask. For one mask and one wearing method, a 4-s video was repeatedly filmed five times, with a total of 100 shots (five types of masks × four wearing methods × repeat five times) taken per person. For the 50 participants, a total of 5000 video data were obtained.

### Models description

Leveraging the temporal dimension of the videos and analyzing changes over time, two primary machine learning-based approaches are available: implicit, involving the extraction of temporal features during the training process of the models, and explicit, where dedicated modules are responsible for extracting temporal features^33^. This study developed both an implicit approach, utilizing a 3D Convolutional Neural Network (3DCNN), and an explicit approach, employing Convolutional Long Short-Term Memory (ConvLSTM), to learn spatiotemporal features for video analysis^34,35^. The 3DCNN utilizes neural network architecture that conducts convolution operations across three dimensions (height, width, depth), while the ConvLSTM combines the spatial feature extraction of CNN with the time series analysis of LSTM. Detailed fundamental structural features of each model are available in Supplementary Fig. S1 and S2. Earlier studies have demonstrated their effectiveness in diverse tasks, including motion recognition^36^, large-scale video behavior classification^37^, and weather prediction^38,39^. These research studies explored deep learning models such as Spatial Pyramid Matching, Long-term Recurrent Convolutional Networks, and fully connected LSTM. Nevertheless, they demonstrated the superior effectiveness of 3DCNN and ConvLSTM in analyzing video data^36–38^. This is attributed to their ability to exploit spatial and temporal dimensions within the videos. Therefore, this study developed efficient models founded on these architectures to analyze breathing patterns in videos captured after wearing a mask, leveraging their ability to learn temporal flow and spatial image characteristics.

The proposed models were developed from scratch, with starting weights randomly initialized. Designing models from the ground up enabled this study to explore structures and parameters carefully adjusted to the characteristics of the specific dataset, achieving satisfactory classification performances while maintaining computational efficiency. Random weight initialization involves commencing the model with weights set to random values, a technique aiding the model in learning inherent patterns during training. This prevents overfitting while enhancing the model's generalization capabilities. Although this study attempted to utilize well-known pre-trained models such as VGG-16 and ResNet, the characteristics of the thermal video data posed challenges for efficient model learning. The training duration for both proposed models is comparable, approximately 9 h. As for inference time, the 3DCNN and ConvLSTM models demonstrate a range between 2 and 3 s.

### 3DCNN

**Figure 2:**
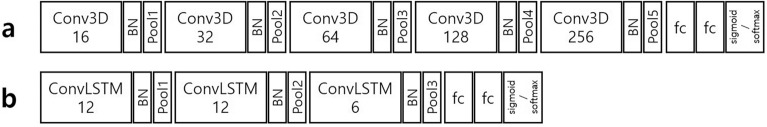
Representation of the proposed models. (**a**) The 3DCNN architecture; (**b**) the ConvLSTM architecture.

The architecture comprises five 3D convolutional layers, individually followed by batch normalization, a rectified linear unit (ReLU), and a pooling layer. Each convolutional layer utilizes a 3 × 3 × 3 kernel size, a stride of 1, and zero padding to ensure comprehensive feature extraction from the input volume. Following batch normalization, ReLU serves as the activation function within the fully connected layer, introducing non-linearity to the process. The number of filters progressively increases, starting from 16 and reaching up to 256. Both max pooling and average pooling operations are employed to reduce the dimensionality of the feature maps, preventing overfitting while enhancing computational efficiency, with a kernel size of 2 × 2 × 2 and a stride of 2. Towards the end of the architecture, a flattening layer is included, followed by a fully connected dense layer with 100 nodes and dropout for regularization. The output layer includes a sigmoid activation function for binary classification and a softmax for multi-classification. The model utilizes the Adam optimizer with a learning rate 0.0001 and an L2 regularization strength of 0.1, applied to all weights to prevent overfitting. For binary classification, the 3DCNN model in this study contains 3,687,817 parameters; for multi-classification, it has 3,688,120 parameters. The architecture of the 3DCNN used in this study is visualized in Fig. 2a.

### ConvLSTM

The model initiates with a sequence of ConvLSTM layers, each featuring 12 filters and a kernel size of 3 × 3, except for the final ConvLSTM layer, which encompasses 6 filters. These layers are designed to maintain the temporal sequence of data, with the initial two returning sequences and the last one consolidating the temporal information. To mitigate overfitting, L2 regularization with a strength of 0.1 is applied to all convolutional layers. Following each ConvLSTM layer, batch normalization and a ReLU activation function are employed, mirroring the structure of the previous model. Spatial dimension reduction is achieved through max pooling layers after the initial two ConvLSTM layers, while an average pooling layer follows the last ConvLSTM layer. All pooling layers have a stride of 2. The model then transitions to a flattening layer, converting the 3D feature maps into a 1D vector. This is followed by a dense layer comprising 100 nodes, succeeded by a dropout layer with a rate of 0.5. The output layer and optimizer retain the same configuration as the previously described model. The ConvLSTM model comprises 491,577 parameters for binary classification and 491,880 for multi-classification. The architecture employed in the ConvLSTM is depicted in Fig. 2b.

### Data analysis

**Figure 3:**
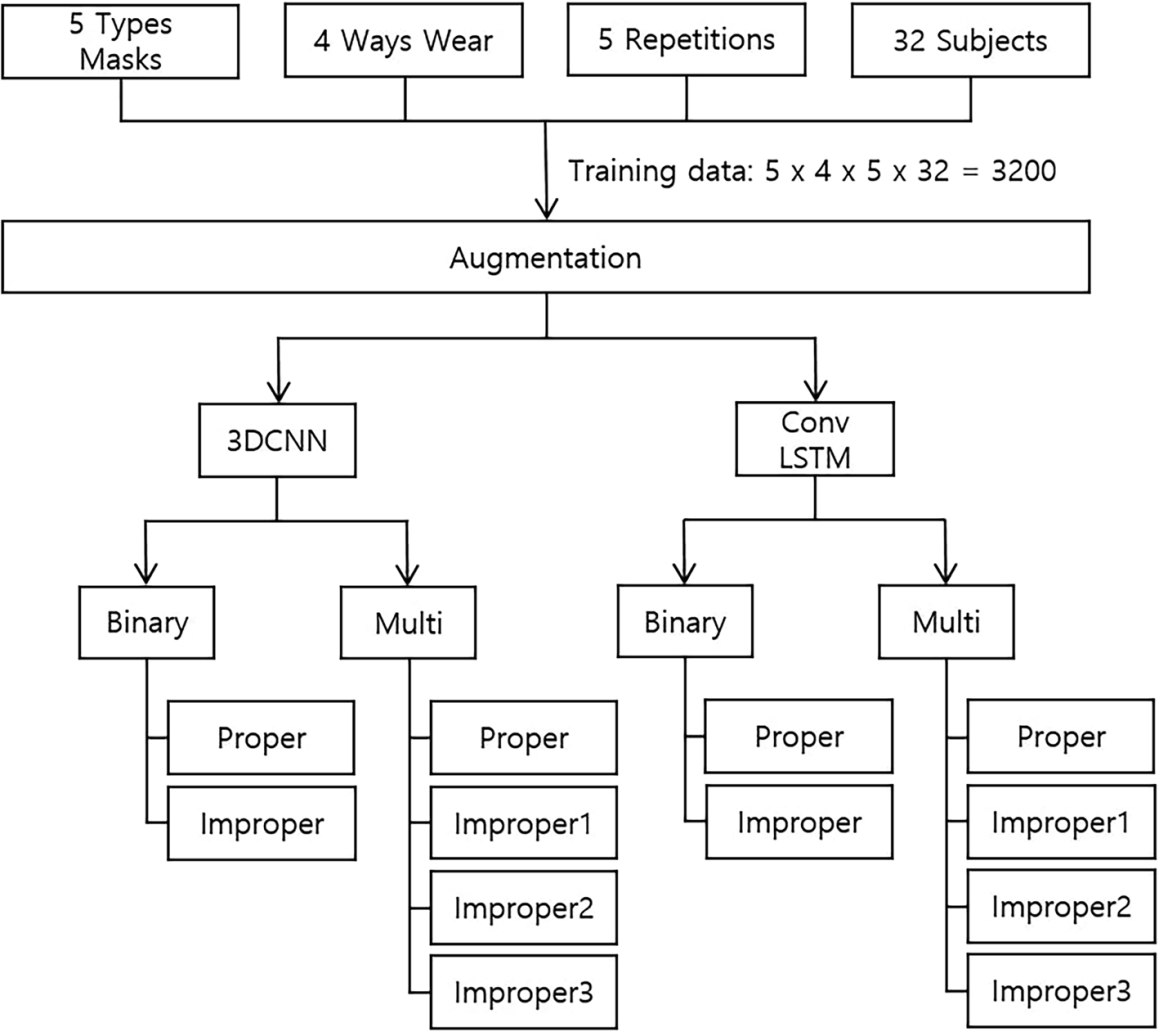
Overview of the training process.

Of the 50 participants, 32, 8, and 10 were allocated to the training, validation, and test sets, respectively. We separated the participants for each set to avoid duplicating the same participants across sets. Figure 3 shows the analytical process used for analyzing the training set. Factors that differentiated the data included five mask types, four wearing methods, five repeated shots, and different participants. Eight types of image data augmentation were randomly applied before the learning process. The specific methods employed are detailed in Supplementary Table S1^40^. A binary classification approach was developed employing 3DCNN and ConvLSTM to differentiate between correct (Fig. 1D) and incorrect (Fig. 1A–C) mask-wearing methods. A multi-classification approach was also developed to categorize the four distinct wearing methods (Fig. 1A–D). The classification of wearing a mask correctly was denoted as "proper," while the other methods were as "improper."

### Metrics

Accuracy, area under the receiver operating characteristics curve (AUROC), precision, recall, specificity, and F1-score were used as model performance evaluation indicators. Accuracy is the simplest and most intuitive measure and is calculated by dividing the number of correct predictions by the total number of predictions. It was chosen because it gives a quick and straightforward overview of the model’s overall performance. AUROC is a value obtained by graphing the true-positive rate (TPR) against the false-positive rate (FPR) at various thresholds and by calculating the area under it. It is used because it provides an aggregated measure of performance across all possible classification thresholds, thus reflecting the model’s ability to distinguish between classes. Precision is the proportion of predicted positive cases that are actually positive. This metric was selected to understand how many positively predicted cases were positive, reducing the risk of false positives. Recall indicates the proportion of positive cases predicted as positive, which is crucial for ensuring the model's capability to identify the majority of positive instances. Specificity, the ratio of predicted negative cases that are actually negative, is crucial for measuring the model's accuracy in correctly identifying negative cases, especially in situations where false positives can have significant consequences. The F1-score, derived as the harmonic average of recall and precision, served as a performance evaluation metric, with higher values signifying superior results. This measure is especially valuable in domains like the medical field, where unbalanced classification is prevalent, and achieving a balance between precision and recall is critical. For the assessment of the multi-classification model, a micro-average approach was employed to calculate precision, recall, AUROC, and F1-score.

### Software and Hardware specifications

The analysis was performed on an Ubuntu 16.04.7 LTS server with 500 GB of memory. The server runs on 48 CPUs, particularly the Intel(R) Xeon(R) CPU E5-2687W v4 model, clocked at 3.00 GHz. It has 8 NVIDIA Tesla P100 PCIe GPUs for graphical processing, each with 16 GB of memory. The software environment is managed by conda version 4.9.2, and the analysis uses Python version 3.8.2 in conjunction with TensorFlow version 2.3.1.

### Ethics declarations

This study was conducted in full accordance with all relevant guidelines and regulations. The study was approved by the Institutional Review Board of The Catholic University of Korea, Seongeui Medical Campus (MC23OISI0006). All participants provided written informed consent. The consent form explained the purpose of the study, the risks and benefits of participating, and the participants' right to withdraw from the study at any time. The study has been performed in accordance with the declaration of Helsinki.

## Results

### Participant characteristics

Table 1 shows participants' demographics and clinical data in infrared video recording. The 50 participants included 39 women (78%) and 11 men (22%) with an age range of 22 to 60 years (median = 35.5 years, SE = 1.59), and the mean age was 38.04 years (SD = 1.59). The height of participants ranged from 153 to 184 cm (mean = 164.86 cm, SD = 8.07; median = 162 cm, SE = 1.1), their weight ranged from 42 to 93 kg (mean = 59.36 kg, SD = 11.23; median = 58 kg, SE = 1.59), and their BMI ranged from 17.16 to 29.69 kg/m^2^ (mean = 21.71 kg/m^2^, SD = 2.86; median = 21.25 kg/m^2^, SE = 0.4).

**Table 1:** Demographics and clinical data of participants (*n* = 50).

Characteristics	F	%		
Sex				
Male	11	22		
Female	39	78		
Characteristics	Mean	SD	Median	SE
Age (years)	38.04	11.22	35.50	1.59
Height (cm)	164.86	8.07	162.00	1.14
Weight (kg)	59.36	11.23	58.00	1.59
BMI (kg/m^2^)	21.71	2.86	21.25	0.40

### Performance of the classification models

Table 2 presents the experimental results of 3DCNN and ConvLSTM, derived from the analysis of all 1000 test data. In the binary classification task, 3DCNN exhibited superior performance, with accuracy, AUROC, precision, recall, specificity, and F1-score values of 0.934, 0.982, 0.829, 0.928, 0.936, and 0.876, respectively, outperforming ConvLSTM in all metrics. In the multi-classification task, 3DCNN consistently outperformed ConvLSTM across various metrics. Notably, the accuracy, AUROC, precision, recall, specificity, and F1-score for 3DCNN were 0.875, 0.986, 0.875, 0.875, 0.958, and 0.875, respectively. In binary classification, 3DCNN achieved a higher accuracy of 0.934 compared to ConvLSTM's 0.908. Similarly, in multi-classification, 3DCNN demonstrated an accuracy of 0.875, surpassing ConvLSTM's 0.862. It is noteworthy that binary classification exhibited superior accuracy compared to multi-classification. The AUROC value for multi-classification was notably high at 0.986, indicating the model's ability in binary and multi-class classification. Supplementary Fig. S3 illustrates the ROC curves for each classification.

**Table 2:** Results of using all test sets (*n* = 1000).

Classification type	Model	Accuracy	AUROC	Precision	Recall	Specificity	F1-score
Binary	3DCNN	0.934	0.982	0.829	0.928	0.936	0.876
	ConvLSTM	0.908	0.977	0.767	0.908	0.908	0.832
Multi	3DCNN	0.875	0.986	0.875	0.875	0.958	0.875
	ConvLSTM	0.862	0.976	0.862	0.862	0.954	0.862

Table 3 presents the classification results derived from 3DCNN based on mask types. Irrespective of the classification type, all mask types demonstrated AUROC values exceeding 0.9. Among them, the KF-AD mask achieved the highest AUROC value of 0.997 in binary classification, indicating superior classification performance. In binary classification, the accuracy, precision, recall, specificity, and F1-score for KF-AD were 0.955, 0.848, 1, 0.94, and 0.917, respectively. In multi-classification, KF-AD exhibited an accuracy of 0.955, AUROC of 0.995, precision of 0.955, recall of 0.955, specificity of 0.985, and F1-score of 0.955. KF-AD and KF94 masks achieved a recall value of 1 in binary classification and scored 0.9 or higher in accuracy, AUROC, recall, specificity, and F1-score for binary and multi-classification. KF80 also performed exceptionally well, with a high AUROC of 0.996 in binary and multi-classification, scoring 0.9 or higher on all other evaluation metrics. Compared to other masks, KF99 was relatively less accurately classified.

**Table 3:** Results by mask type using 3DCNN (*n* = 200 per mask type).

Classification type	Mask type	Accuracy	AUROC	Precision	Recall	Specificity	F1-score
Binary	Surgical mask	0.905	0.976	0.746	0.940	0.893	0.832
	KF-AD	0.955	0.997	0.848	1.000	0.940	0.917
	KF80	0.965	0.996	0.906	0.960	0.967	0.932
	KF94	0.955	0.988	0.848	1.000	0.940	0.917
	KF99	0.890	0.938	0.804	0.740	0.940	0.771
Multi	Surgical mask	0.835	0.977	0.835	0.835	0.945	0.835
	KF-AD	0.955	0.995	0.955	0.955	0.985	0.955
	KF80	0.945	0.996	0.945	0.945	0.982	0.945
	KF94	0.910	0.989	0.910	0.910	0.970	0.910
	KF99	0.730	0.959	0.730	0.730	0.910	0.730

### Comparison of prediction results according to the mask-wearing method

**Figure 4:**
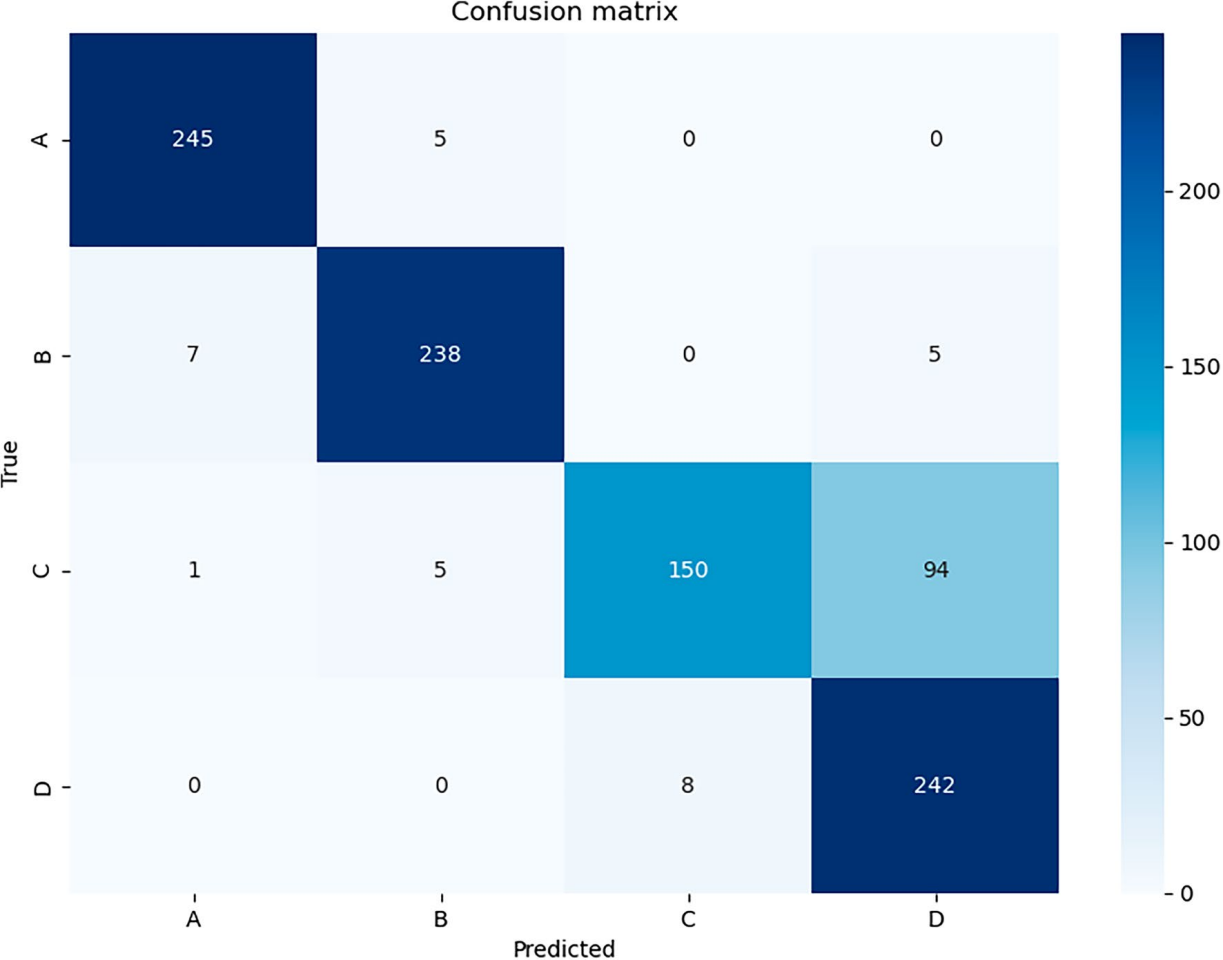
Confusion matrix (4 × 4) of multi-classification in 3DCNN. (**A**) Wearing a mask on the chin to expose the nose and mouth; (**B**) lowering the mask under the nose and covering only the mouth; (**C**) covering the nose without the nose wire being tightly attached, thereby letting air leak from the side of the mask; (**D**) wearing the mask properly and securely.

The confusion matrix in Fig. 4 details the results of multi-classification using 3DCNN. The matrix contains various mask-wearing methods, denoted as A, B, C, and D, as depicted in Fig. 1, with each category comprising 250 data points. Method A exhibited the highest accuracy, achieving a correct prediction rate of 98%. Similarly, methods B and D demonstrated high accuracy, with correct prediction rates of 95.2% and 96.8%, respectively. However, method C had the lowest accuracy, with a prediction accuracy of only 60%. Notably, the most frequent misclassification for method C occurred as method D, underscoring the challenge of distinguishing between these mask-wearing methods.

## Discussion

Preventing the inhalation and spread of particulate matter, known to be harmful to human health^41^, is essential. Moreover, infection prevention has consistently been a significant concern, with an intensified focus on safeguarding healthcare workers at high risk of exposure, particularly amid the COVID-19 pandemic^42,43^. Masks serve as a crucial means of protection against these risks.

This study proposed an improved approach utilizing infrared video captured by a thermal imaging camera and leveraging deep learning techniques to identify the correct wearing of masks accurately. The study incorporated five types of masks (surgical mask, KF-AD, KF80, KF94, and KF99) recommended for diverse environments, including hospitals. The research utilized 3DCNN and ConvLSTM models to classify three improper mask-wearing methods and one proper mask-wearing method, serving the healthcare workers and other individuals vulnerable to associated risks.

In summary, this study revealed the superior performance of 3DCNN in binary and multi-classification tasks. The model's AUROC score of 0.986 signifies its high accuracy in providing correct predictions with a low rate of false positives. This underscores the efficacy of 3DCNN in distinguishing between proper and improper mask-wearing methods. Across all types of masks, the AUROC value exceeded 0.9, indicating the model's capability to classify correct mask-wearing irrespective of the type. The mask that achieved the highest classification performance was KF-AD, with most of its scores surpassing 0.9. Specifically, the recall value in binary classification was 1, signifying that the model could flawlessly identify the correct mask-wearing instances for KF-AD. Additionally, KF94 exhibited a recall value of 1 and demonstrated values of 0.9 or higher in accuracy, AUROC, and F1-score, indicating excellent overall performance. Moreover, surgical masks and KF80 exhibited respectable classification performance, whereas KF99 showed relatively lower classification performance. The observed differences in classification performance may be attributed to the design variation. In contrast to other KF masks that adopt a horizontal folding approach, the KF99 is designed with a vertical fold. The strict adherence to regulations and use of a thicker and more rigid material in KF99 resulted in a less secure attachment to the face. Consequently, the vertically folded design made it easier for air to leak through the back of the mask, especially when the mask is slightly larger than the face^44^. Improved results could be expected with a more customized mask selection based on the participant's face shape. Other mask types consistently achieved high scores across all metrics, accurately recognizing correct mask-wearing. Moreover, the model demonstrated robust performance in multi-classification, showcasing its ability to distinguish various wearing methods and enhance reliability and validity, which is particularly valuable as individual mask-wearing behavior varies from completely improper to partially improper.

**Figure 5:**
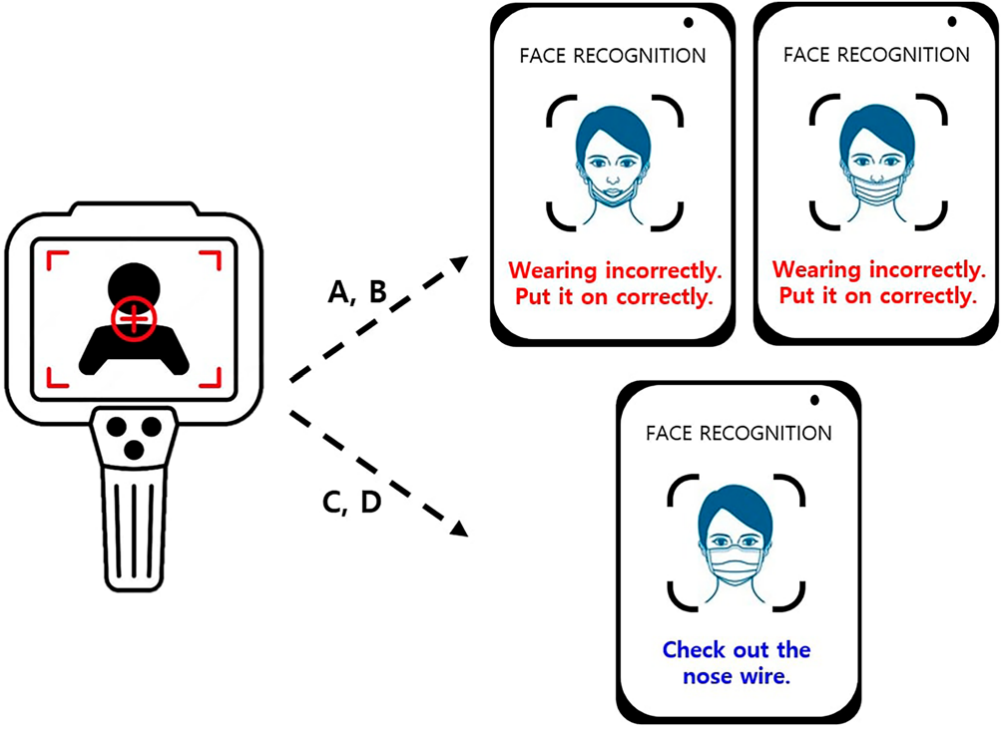
Examples of real-world application of the device for determining proper mask fit. (**A**) Wearing a mask on the chin to expose the nose and mouth; (**B**) lowering the mask under the nose and covering only the mouth; (**C**) covering the nose without the nose wire being tightly attached, thereby letting air leak from the side of the mask; (**D**) wearing the mask properly and securely.

The confusion matrix generated from the multi-classification using 3DCNN effectively differentiated between mask-wearing methods A, B, and D. Methods A and B involve masks covering a smaller facial area, facilitating clear discrimination between improper methods and the correct wearing method. However, method C was occasionally misclassified as D, possibly due to facial shapes allowing minimal air leakage even when the nose wire was not tightly secured, resulting in a subtle temperature difference. While this might not pose a significant issue in practical use. Providing instructions to wear the mask properly upon detecting methods A or B and advising a check of the nose wire for methods C or D could effectively ensure the safety of mask wearers in real-world scenarios (Fig. 5). Such instructions could have positive implications for both medical and occupational health.

This study introduces a method for swiftly and efficiently assessing improper mask-wearing in real-time, significantly improving over existing time-consuming and impractical methods. Unlike traditional approaches, where costs per test rise^17^, the expenses in this method might remain unaffected by the number of test participants, provided that only a thermal imaging camera is available. Consequently, this research holds critical implications for occupational settings with elevated risks of exposure to harmful particles and infectious diseases, particularly in fields like healthcare. In environments where mask-wearing is essential, the accurate real-time recognition of wearer behavior through a thermal imaging camera can safeguard workers' health and well-being. This technology benefits people in diverse settings, including healthcare, by ensuring proper mask usage, mitigating exposure to harmful elements, and enabling a safer work environment. Further research and development are necessary to propose and verify improved methods, exploring their potential application in high-risk environments with severe fine dust and pollutants exposure.

This study has several limitations. Firstly, the results may not be widely applicable across different races, sexes, and ages, as it only included Asian participants and a small number of males. This limitation could be mitigated by employing a thermal imaging camera that focuses on heat emission characteristics related to body temperature rather than surface characteristics like skin color. Additionally, the methodology of capturing multiple videos from each participant could potentially inflate the results, limiting diversity and diminishing the objectivity of the findings. The repeated appearance of the same participants within each set may introduce bias in assessing the model's generalizability. Factors such as environmental congestion, air circulation, temperature, and humidity at the data collection sites could influence the performance of thermal imaging cameras. Moreover, in real-world scenarios, additional variables might be present beyond the type of mask or mask-wearing method covered in this study. Lastly, the current proposed model is relatively unreliable in distinguishing between properly fitted and slightly looser methods, indicating a need to explore applications that can be more effectively integrated.

Furthermore, we need to mention that face detection methods were employed on the video frames within the dataset as a preprocessing step. Nevertheless, these methods faced challenges in consistently detecting faces, primarily attributed to the indistinct features of eyes, nose, and mouth in thermal images. Utilizing facial detection techniques specifically designed for thermal images has the potential to enhance the effectiveness of recognizing mask-wearing status, regardless of the distance between the camera and the person. In future studies, addressing these limitations could involve expanding the scope by incorporating larger sample sizes and diverse types of masks in various settings and environments. This approach would contribute to a more comprehensive and accurate verification process of proper mask fitting.

## Conclusions

This study developed an artificial intelligence system using thermal imaging technology to monitor mask-wearing methods efficiently. This system can quickly and cost-effectively detect temperature changes in any mask type. Its applications in hospitals, quarantines, and workplaces offer real-time identification of improper mask use, enabling timely intervention. This innovation could enhance safety and health-promotion effectiveness in various settings while laying the foundation for future sophisticated technology for mask-wearing behavior monitoring. The study contributes to better healthcare environments, improved public and occupational health, and increased mask-wearing compliance. Strategies can be further explored to transcend the limitations imposed by a single racial environment.

## Supplementary Information

Supplementary Information.

## Data Availability

The video datasets generated during the current study were deposited into public repository (https://doi.org/10.5281/zenodo.10244188).
