# Supplementary material for: Thermal imaging and deep learning-based fit-checking for respiratory protection
Source: Sci Rep. 2024 Oct 17;14:24407. doi: 10.1038/s41598-024-52999-0 (PMC11487064; doi:10.1038/s41598-024-52999-0)
Supplement: Supplementary file 1 — Supplementary Information. [file 41598_2024_52999_MOESM1_ESM.pdf]

Research Article

Thermal Imaging and Deep Learning-based Fit-checking for Respiratory Protection

Hyunjin Kim[1,2], Tong Min Kim[2], Sae Won Choi[4], Taehoon Ko[1,2,3*]

[1]*Department of Biomedicine & Health Sciences, College of Medicine, The Catholic University of Korea, 222 Banpo-daero, Seocho-gu, Seoul, Republic of Korea*

[2]*Department of Medical Informatics, College of Medicine, The Catholic University of Korea, 222 Banpo-daero, Seocho-gu, Seoul, Republic of Korea*

[3]*CMC Institute for Basic Medical Science, the Catholic Medical Center of The Catholic University of Korea, 222 Banpo-daero, Seocho-gu, Seoul, Republic of Korea*

[4]*Office of Hospital Information, Seoul National University Hospital, 101 Daehak-ro, Jongno-gu, Seoul, Republic of Korea*

***Corresponding Author: Taehoon Ko, email:** thko@catholic.ac.kr.

Authors' email addresses: Hyunjin Kim, hyunjinkim9729@gmail.com; Tong Min Kim, dianakim@catholic.ac.kr; Sae Won Choi, swc@snuh.org; Taehoon Ko, thko@catholic.ac.kr;

Supplementary Figure 1. Basic 3DCNN description

The 3DCNN is an extension of the 2D convolutional neural network (2DCNN), which is commonly used for video-processing tasks; moreover, whereas 2DCNN receives 2-dimensional input (height and width), 3DCNN is designed to process 3-dimensional data (height, width, and depth) and is characterized by the ability to learn spatiotemporal features directly from the input [1, 2]. The filter of the convolution layer is three-dimensional and detects patterns by sliding according to the input data's height, width, and depth. The pooling layer reduces the dimensionality of the output to decrease complex calculations and prevent overfitting. The fully connected layer takes previously learned high-level features and performs final tasks like classification or regression [3]. The figure below shows the basic structure of the 3DCNN.

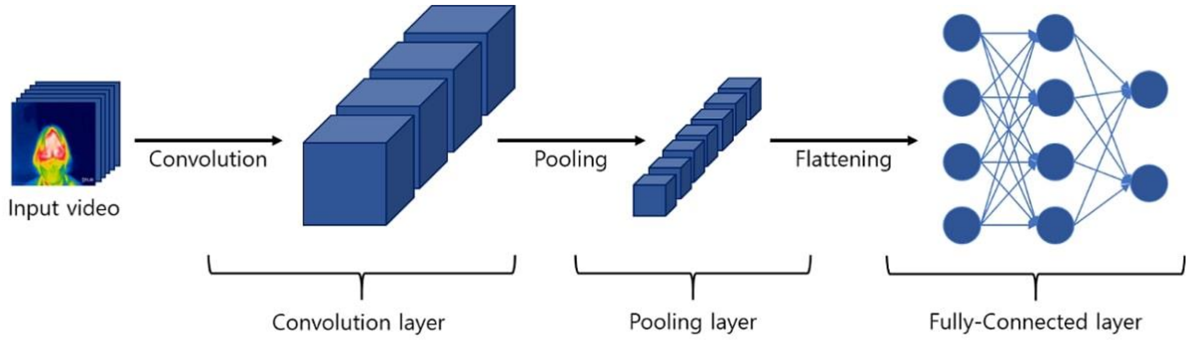

Basic 3DCNN architecture.

Supplementary Figure 2. Basic ConvLSTM description

ConvLSTM is a type of recurrent neural network (RNN) that sequentially processes spatiotemporal properties of video by using convolution, instead of matrix multiplication, in the network [4]. As a modification of the existing long short-term memory (LSTM), ConvLSTM is characterized by the addition of a convolution layer to the input sequence to effectively learn the spatial information of the frame image [5]. Moreover, as time information can be considered through repetitive LSTM operations, the LSTM is suitable for video processing and weather prediction [6]. The figure below shows the inner structure of ConvLSTM.

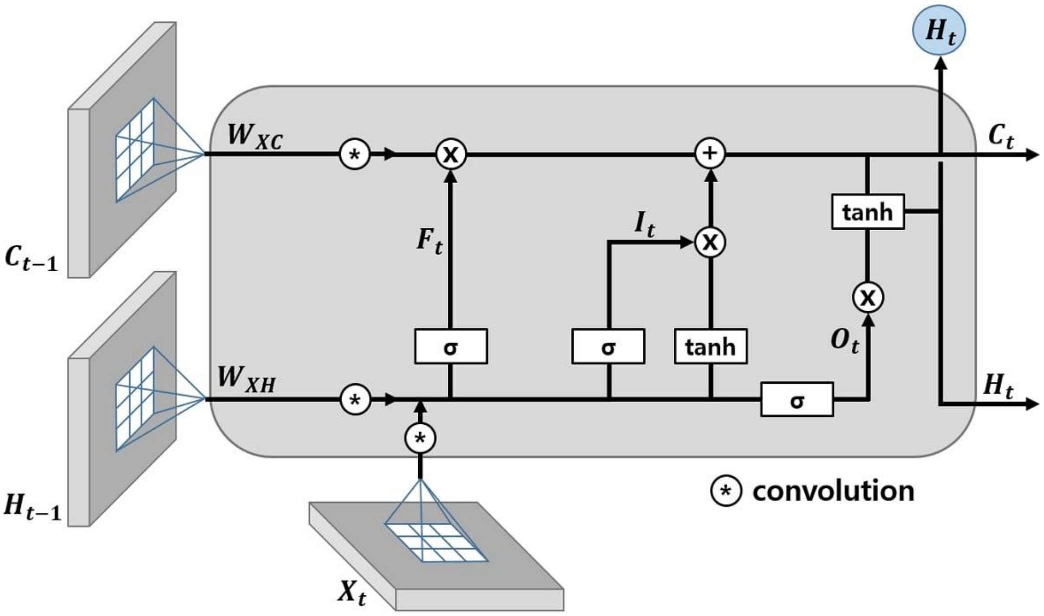

Inner structure of the ConvLSTM.

Supplementary Figure 3. ROC curves for each classification

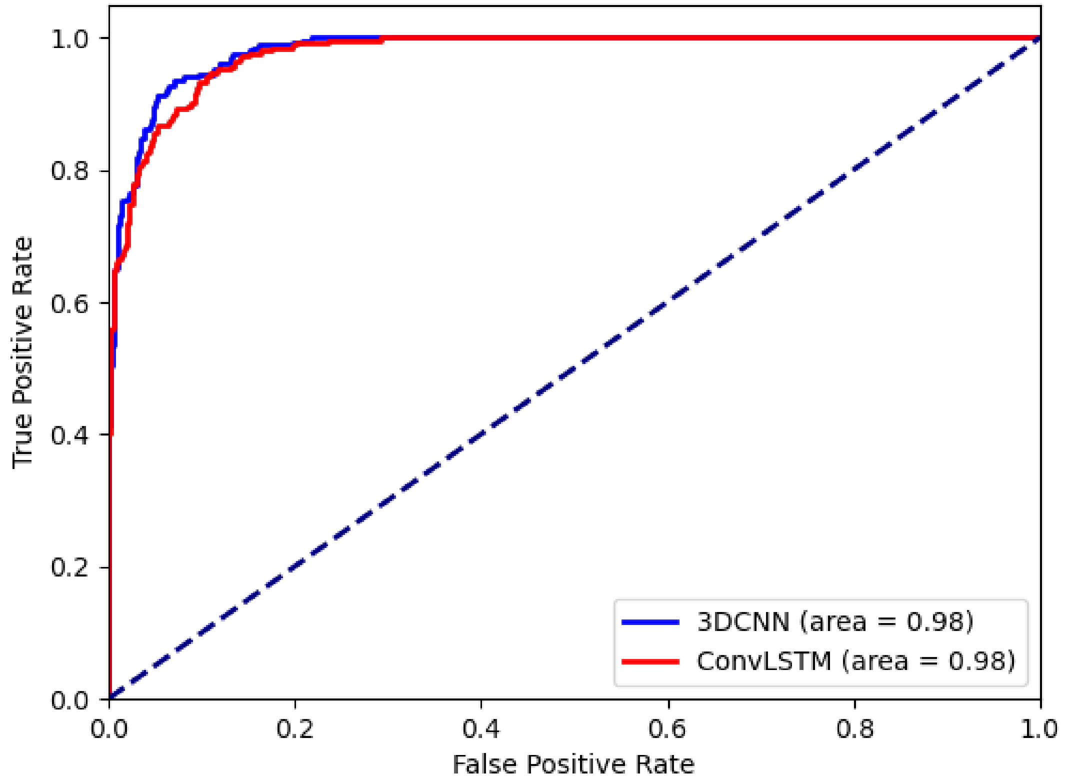

ROC curve for binary classification.

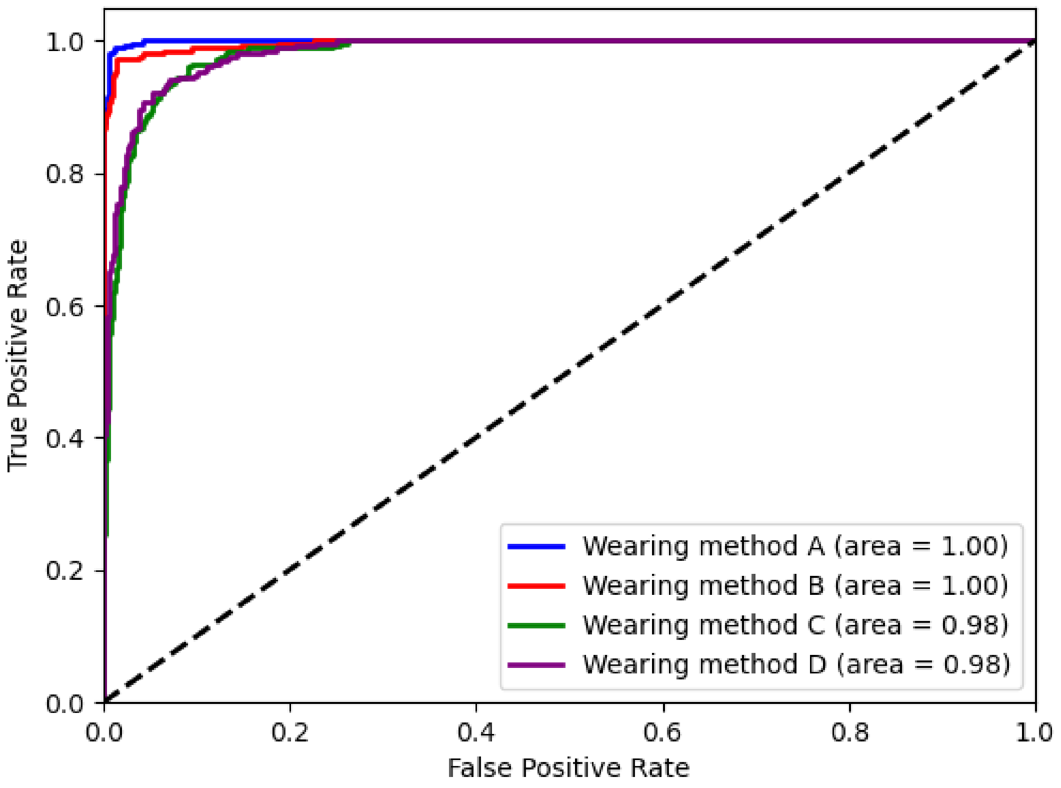

ROC curve for multi-classification using 3DCNN.

This plot curves the ROC value with the corresponding wearing method set to positive and the rest set to negative. Wearing methods are as follows: **A** wearing a mask on the chin to expose the nose and mouth; **B** lowering the mask under the nose and covering only the mouth; **C** covering the nose without the nose wire being tightly attached, thereby letting air leak from the side of the mask; **D** wearing the mask properly and securely.

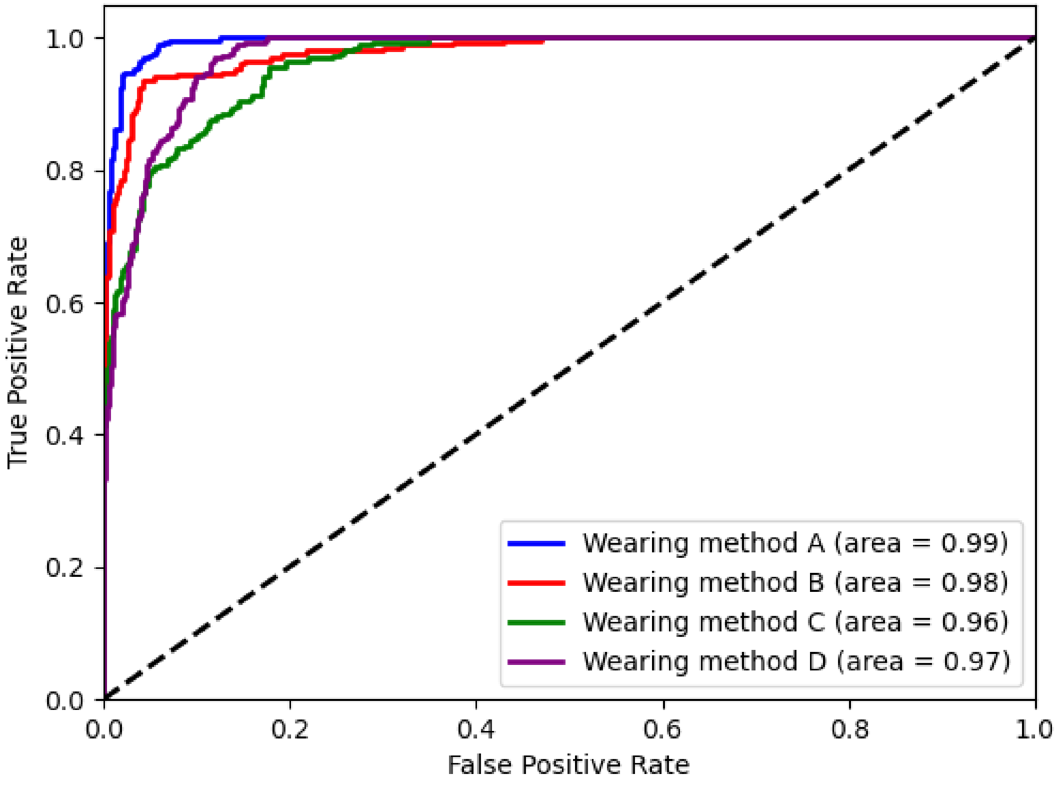

ROC curve for multi-classification using ConvLSTM.

This plot curves the ROC value with the corresponding wearing method set to positive and the rest set to negative. Wearing methods are as follows: **A** wearing a mask on the chin to expose the nose and mouth; **B** lowering the mask under the nose and covering only the mouth; **C** covering the nose without the nose wire being tightly attached, thereby letting air leak from the side of the mask; **D** wearing the mask properly and securely.

Supplementary Table 1. Data augmentation operations

Types	Description
Horizontal flip	Horizontally flip video
Shear	Shearing video in X and Y directions randomly selected from [−0.15, +0.15]
Translate	Shifting video to X and Y coordinates randomly selected from [−50, +50]
Elastic transformation	Field distortion with a strength of 1.5
Multiply	Multiply all pixel intensities by 1.5
Add	Adds −30 to all pixel intensities
Salt	Determines white pixels in each frame with a ratio of 15
Pepper	Determines black pixels in each frame with a ratio of 15

Supplementary Table 2. Experimented architectural components and hyperparameters in 3DCNN

Parameters	Candidates
Number of convolutional layers	3, 4, 5, 6
Filter	5, 6, 8, 10, 12, 16, 32, 64, 128, 256
Pooling	Max, Average, Global average
Lerning rate	0.0001, 0.0005, 0.001
Regularization	None, L1, L2
Strength of regularization	0.01, 0.03, 0.05, 0.1
Dropout rate	0.3, 0.5
Kernel size	3, 5
Stride	1, 2
Batch size	24, 32, 40

Supplementary Table 3. Experimented architectural components and hyperparameters in ConvLSTM

Parameters	Candidates
Number of hidden layers	2, 3, 4
Filter	5, 6, 8, 10, 12, 16
Pooling	Max, Average, Global average
Lerning rate	0.0001, 0.0005, 0.001
Regularization	None, L1, L2
Strength of regularization	0.01, 0.03, 0.05, 0.1
Dropout rate	0.3, 0.5
Kernel size	3, 5
Stride	1, 2
Batch size	16, 24, 32

REFERENCES

[1] Ji, S., Yang, M. & Yu, K. 3D convolutional neural networks for human action recognition. IEEE Trans. Pattern Anal. Mach. Intell. **35**, 221–231 (2013).

[2] Tran, D. et al. Learning spatiotemporal features with 3D convolutional networks. In Proceedings of the IEEE International Conference on Computer Vision, 4489–4497 (2015).

[3] Ahmed, T., Parvin, M.S., Haque, M.R. & Uddin, M.S. Lung cancer detection using CT image based on 3D convolutional neural network. J. Comput. Commun. **8**, 35 (2020).

[4] Shi, X. et al. Convolutional LSTM network: A machine learning approach for precipitation nowcasting. In Cortes, C. et al. (eds) Advances in Neural Information Processing Systems. vol. 28, 802–810 (Curran Associates, Inc, 2015).

[5] Shi, C. et al. Learning multiscale temporal-spatial-spectral features via a multipath convolutional LSTM neural network for change detection with hyperspectral images. IEEE Trans. Geosci. Remote Sens. **60**, 1–16 (2022).

[6] Wu, M. Sequential images prediction using convolutional LSTM with application in precipitation Nowcasting (Master's thesis, Science). University of Calgary (2019). https://prism.ucalgary.ca/server/api/core/bitstreams/3310a71e-b151-4c64-bafc-c4b2b5af50d0/content (Accessed: 19th May 2023).
